# Reflective capacity and context of reflections: qualitative study of second-year medical students’ learning diaries related to a general practice course

**DOI:** 10.1186/s12909-024-05199-3

**Published:** 2024-03-01

**Authors:** Elina Paloniemi, Maria Hagnäs, Ilona Mikkola, Markku Timonen, Ritva Vatjus

**Affiliations:** 1 Research Unit of Population Health, University of Oulu, Oulu, Finland; 2 Rovaniemi Health Center, The Wellbeing Services County of Lapland, Rovaniemi, Finland

**Keywords:** Medical education, Empathy, Self-reflection, Medical curriculum, Undergraduate

## Abstract

**Background:**

Reflective capacity is a prerequisite for transformative learning. It is regarded as an essential skill in professional competence in the field of medicine. Our aim was to investigate the reflective capacity and the objects of action (themes) which revealed reflective writing of medical students during a general practice/family medicine course.

**Methods:**

Second-year medical students were requested to write learning diaries during a compulsory course in general practice/family medicine consisting of the principles of the physician-patient relationship. The course included a group session supervised by a clinical lecturer and a 3-day training period in a local health centre. We conducted data-driven content analysis of the learning diaries. In the learning diaries, student observations were most commonly directed to events during the training period and to group sessions. Occasionally, observation was directed at inner experience.

**Results:**

The following themes were related to reflective writing: feelings towards the end of life, demanding situations in practice, physician’s attitude to patient, student’s inner experiences, and physician’s well-being. The entries indicated different types of reflective capacity. Three subgroups were identified: ‘simple reporting,’ ‘reflective writing,’ and ‘advanced reflective writing.’

**Conclusion:**

Professional growth requires the development of reflective capacity, as it is essential for successful patient care and better clinical outcomes. To develop and enhance the reflective capacity of medical students during their education, the curriculum should provide frequent opportunities for students to assess and reflect upon their various learning experiences.

## Background

Reflection is regarded as an essential professional competence in the field of medicine [1, 2] and has been defined in various ways, but many of these definitions remain unclear or incomplete. Reflection can be divided into two types, namely scientific reflection and personal reflection. Scientific reflection is primarily oriented towards clinical reasoning and existing literature in order to make better, evidence-based clinical judgements [3]. Personal reflection, by comparison, can be defined as ‘the careful exploration and appraisal of experience, thus clarifying and creating meaning for the benefit of balanced functioning, learning and development’ [4]. Reflective ability can be considered as rethinking experiences (both one’s own and those of others) to make decisions about behaviour [5].

Reflections and related utterances can be interpreted using dialogical sequence analysis (DSA) [6], which is a microanalytic method based on the theory of Mikhail Bakhtin [7] and Vygotskyan [8] sign-mediated activity. A person’s actions have an object and they always relate to the object in some way. Even mental activity has an object. People’s attitudes towards objects can be seen in their expressions when objects are addressed [8]: ‘When communicating, individuals simultaneously position themselves with regard to the referential object and the addressee. Depending “about what” people are speaking and “to whom” they direct their words affect the style and composition of their utterances’ [6].

Critical reflection can be considered as critical thinking about the prejudices on which our beliefs are built. Mesirow defines learning as ‘the process of making a new or revised interpretation of the meaning of an experience, which guides subsequent understanding, appreciation and action’ [9]. Critical reflection is a requirement for transformative learning [9], and the process and practice of reflection enhance learning during medical education [10, 11] and may improve self-understanding [12]. These also lead to improvement in the levels of empathy and respect for patients, the ability to practise self-reflection, clinical and cultural competencies, communication skills and continual professional development [10, 11].

Reflective writing (e.g., using a portfolio or learning diary or writing essays) is commonly used to improve and assess students’ reflective capacity during medical education [1, 2, 10, 13]. To the best of our knowledge, no previous studies have examined the reflections and object-related utterances of medical students. Consequently, we conducted a qualitative study to investigate the reflection ability of medical students during a general practice course in the medical curriculum using DSA. The learning diaries of students were investigated to evaluate their reflective ability based on their reflective writing. Additionally, we were interested in the objects of actions (themes) which were revealed during reflective writing.

## Methods

### Research approach

We approached reflection as a phenomenon and utilized an interpretive description [14]. The qualitative analysis of the learning diaries was strongly data-driven, although our baseline interpretation of reflection affected the process. The qualitative content analysis of the learning diaries involved categorizing, thematizing and classifying the material.

When examining the utterance of the students in detail, we adapted semiotic analysis [15]. The final analysis was based on DSA [6], through which an observer can recognize signs and discover the writer’s attitude toward the content of the writer’s utterance.

The research team consisted of the five authors, who are health care professionals and work in the field of medical education. Furthermore, three of the authors have been trained in DSA. Of these three authors, one is also a psychotherapist and another is a psychiatrist. This study was approved by Ethics Committee of the Northern Ostrobothnia Hospital District, Finland (62/2017).

### Context of the study

Medical education in xx universities consists of a 6-year program, of which the first 2 years comprise preclinical studies and are mainly theoretical. General practice / family medicine (GP/FM) is part of the curriculum in all five xx universities providing medical education. At the University of xx, GP/FM takes place every year, and learning diaries are used to facilitate the development of reflective capacity.

In the spring semester of 2019, second-year medical students attended a compulsory course in GP/FM consisting of the principles of the physician-patient relationship. The course focused on interviewing patients, meeting patients with varying backgrounds and challenges, and an overview of multiprofessional teamwork. Specifically, the aim of the course was to guide students to learn appropriate interview techniques (patient/physician-oriented), to understand the patient’s living environment and its significance, and to observe patients and their attitudes towards their disease.

At the beginning of the course, students participated in a compulsory lecture considering the theoretical basics of the issues at hand, including demonstrations of physician- and patient-centred interviews.

After the introductory lecture, students were divided into small groups of 12 or 13. In the group sessions, students practised interviewing patients who expressed strong emotions (e.g., sadness, anger, anxiety or fear) with standardized patients. The situations were similar to real ones, as the standardized patients were highly experienced. Afterwards, students’ experiences were discussed profoundly, and every student received constructive feedback from the supervising clinical lecturer, peer group and standardized patient. A 3-day training period in a local health centre was also included in the course. During the training period, students mainly shadowed general practitioners and GP trainees, and one of the main tasks was to focus on the physician-patient relationship. The training period was the second of its kind and students had had another 3-day practical training period during their first year of study.

### Participants

The participating students entered the University of xx in August 2017 and were invited to the current study then. Of the 154 medical students, 153 gave written consent for the use of collected data for scientific purposes. Of these, 142 finally attended this course and submitted learning diaries, which were included in the analyses. All participants provided written informed consent prior to enrolment in the study.

### Data collection

To improve and assess their reflective capacity, students were asked to complete learning diaries after a compulsory group session and a 3-day practical training period in a local health centre. Instructions for the learning diaries were given during the group session. Appropriate reflective writing was facilitated with the following guided questions *(1) During the group session or practical training period, what factors helped you to reflect on your attitudes, ways of thinking or doing things? (2) During the group session or practical training period, what factors contributed to a sense of self-awareness and role as a physician? (3) What emotions or observations did you detect in yourself when meeting different people?* Students were advised to submit their learning diaries electronically. Learning diaries were collected from the students, read by the researcher and returned with comments to the students. The learning diaries were not collected again.

### Analysis

Analysis of the learning diaries was formed over several readings, and the first researcher read the learning diaries on average seven times. During the initial reading, the first researcher focused on individual students’ experiences, and every student was given short, written feedback (a comment or a short question on their reflection targeted to support them in reflective writing and possibly help them find a new perspective). When the material was read systematically, the focus was on how students described their experiences in their learning diary. Our interpretation of reflection, methodological choices and research findings were clarified through several dialogical conversations.

As analysis continued, it came out that the students indicated different kinds of reflective capacity: at first, five categories were identified by the first researcher according to reflective capacity. The categories were combined, and three final subgroups formed: simple reporting, reflective writing and advanced reflective writing.

Of 142 learning diaries, 125 were categorized directly into three subgroups by the first researcher, and 17 ambiguous samples in all categories were more closely reviewed by the first and last researchers. The last researcher read these 17 samples and independently assigned them to the categories formed and defined by the first researcher. These two researchers agreed on 12 of the 17 samples. However, the differences in interpretations were slight. Finally, the samples were read at a data session together with the research team. This time, the focus was on the few samples the first and the last researcher did not initially agree on. The group eventually agreed on 16 of the 17 samples.

## Results

### Reflective writing and capacity

Practically all the students recognized and described at least some feelings, thoughts and/or attitudes aroused by different situations and experiences during the training period and group session, however reflective capacity varied in the learning diaries. Learning diaries were categorized into three final subgroups: simple reporting, reflective writing and advanced reflective writing.

1. The first group (simple reporting) included learning diaries where the student showed a flash of reflection, but the reflective capacity appeared to be very superficial at this point in their studies. This group remained rather small (*n* = 9). These nine students primarily described what they observed.

*A GP must screen those who really need immediate treatment from an immense number of patients and illnesses.*

*In the case of a substance abuse patient, what was* ***interesting*** *was the discussion between the patient and the doctor about reducing the medication dose, which the patient was opposed to. In this case, the patient used many kinds of methods to ‘prove’ that they needed the current dose.*

The students reported events or their thoughts. In the latter example, the student showed interest in the conversation between the patient and the physician. This can be considered a starting point of reflection, but it remained very superficial, and neither of the students moved on to describe any other feelings, thoughts and/or attitudes that may have arisen in the situations.

2) The second group (reflective writing) included learning diaries where students described feelings, thoughts and/or attitudes towards different situations and experiences. The major part of the learning diaries was categorized into this group (*n* = 87).

*When I think about it in hindsight, the more in-depth discussion with the health centre physician helped me to understand patient work, and I hope the effects will be evident in my work. It also gave me hope that not every patient coming to an appointment is fuming and irritated; the doctor can affect the course of the treatment relationship a lot with his or her own behaviour.*

*During the training period in the health centre I saw a patient****who you might say had problems all over***. *An hour had been reserved for the appointment.… After 30 min, I was already slightly frustrated with the patient’s problems.****It felt like****[she/he] was making up the symptoms described to the doctor. However, the doctor seeing the patient listened and discussed patiently with the patient, although [she/he] later admitted being frustrated during the appointment.*

The difference from the previous samples may seem slight. However, there is a change in the students’ way of expression. In the second group, students start to observe and discuss their thoughts and mindscape (mental or psychological scene or area of the imagination). It is possible to recognize the point (bolded by the authors) where the reader is willing to define the student’s expression and address the Why questions. The reflective conversation might help student understand the importance of observant listening to patients. In the latter example, the student is also able to perceive their self as a separate operator from the patient and physician– they described their own feelings and thoughts but also possible feelings and thoughts of others.

The students in the third group (*n* = 46) (advanced reflective writing) discussed possible reasons behind recognized feelings, thoughts and/or attitudes or showed deeper understanding in other ways.

*… it was particularly hard for me to be neutral towards alcoholics or other groups that I felt had in a way themselves caused many of the problems they had… it was hard to understand why anyone would ‘voluntarily’ drive oneself into situations like that. However, the more patients I saw, the more clearly I was able to see that it’s not likely that anyone had wanted to choose a path that leads to alcohol or drug abuse. For many, their current life situation was the result of a series of unhappy coincidences. For many, the starting point had been challenging right from childhood, and drugs, for example, had been a way to seek relief. When you witnessed various human destinies you came to realize that despite all the equality in our society, it cannot always provide the same starting point for everyone. A lot of things have an effect on how people evolve and what happens to them.*

The student recognized the feeling of unease when they considered the disease or situation to be a consequence of patients’ own choices. They described difficulty understanding those choices. Then, the student described the change in their thinking as they moved on to consider possible reasons behind patient behaviour.

*In this situation, it suddenly dawned on me that as a doctor seeing a patient, it is not my task to change the world so that it is morally to my liking, because another person’s opinion is as right for them as mine is for me.*

In this example, the student recognized the need for a change in one’s thinking and became aware of the importance of a patient-centred approach and a role as a physician.

### Objects of the action (themes) related to reflective writing

Certain experiences and situations during the group session and practical training led to reflective writing; the related utterances reflect the attitudes or reactions of the students. These experiences and situations were regularly mentioned in the learning diaries, although not every theme appeared in every diary. When a student had to deal with a certain kind of object of action in a group session or in practical training, either the chosen words or the expression of a feeling indicated his/her attitude. On the other hand, certain objects that were not mentioned are also worth noting. The following themes induced reflection: end of life, demanding situations in practice, physicians’ attitudes towards patients, students’ own inner experiences, and physicians’ well-being. Below, the themes are described with vignettes. The vignettes are presented in italics, and their interpretations follow.

One of the main themes that induced reflection wasend of life.

*The dying person made me feel a little****sad***, *and I also noticed that it brought back****memories of my grandparents’ passing.***

The student described their feelings and showed self-reflection ability after facing death. They recognized the connection to previous experiences and were able to verbalize it. The student’s utterance showed that they were able to be personally present in this situation.

*During the group session we talked about the approaching death of a patient to the family members with two different doctors […] I noticed that I became a little emotional myself […] I was a bit startled by my own reaction, but on the other hand, it was****understandable, as this was the first time****I was involved in talking about death with family members.*

The student recognized the feeling of sadness and when the patient had an incurable disease. They were startled and able to verbalize their inner dialogue regarding the emotions. Moreover, the student had the metacognitive ability (thinking about one’s own or another’s thinking or feeling) to understand their reaction.

*I noticed feeling****helpless****when the patient was in very poor condition or was generally in a lot of pain.*

*I felt a great sense of****helplessness****when faced with the heavy emotions of an elderly woman who had lost her spouse and suffered from the aftereffects of cancer surgery.*

*And how would I****be able to help****a palliative care patient who is trying very hard to say something but the words don’t come?*

Students described feelings of helplessness when facing human suffering concerning the limitations of life and medicine as well as the existential aspects of life. In the example above, the question itself indicated the feeling.

Another theme repeatedly discussed in the learning diaries was demanding situations in practice.

*It often****feels annoying****to see people who come to the appointment as a result of their own choices, whether it’s smoking, not taking exercise, alcohol or something similar.*

*It was very****frustrating****to see patients who did not take care of their diabetes or themselves at all.*

Students recognized the feelings of annoyance and frustration because of lifestyle diseases and alcohol/drug overuse/abuse. Also, they described their own attitudes towards the diseases they considered to be consequences of the patients’ own choices and towards this group of patients.

*This gave rise to feelings of ‘don’t these people have any regard for their own lives’.*

*A young woman who had previously suffered from childlessness but had got pregnant without meaning to do so and who now wanted to have an abortion gave rise to****strong feelings and questions.***

In the following example, the studentwere getting closer to the ability to reflect on why those people did not seem to care about themselves. The attitude towards patients was not as hard as in the previous examples, and the students showed an ability to understand the diversity of life.

*Heavy service users made me ponder how you should treat patients who come to the clinic very often, always with a different complaint, or on the hand, patients who come in every week with the same complaint, and if there is no medical explanation for their problems.*

Here, the student described thoughts aroused after facing patients suffering from psychosomatic symptoms. They are considering the basis of general practice– these patients are challenging for experienced physicians, too.

*Previously, I might perhaps think that patients consider health and how to promote it as the most important thing in life. This is probably because personally, I consider my own health extremely important. Now I see that patients can have things that they consider more important and even more pressing. For example, the patient may be really rushed or under a lot or stress at work, which is why taking care of one’s health gets less attention.*

The student recognized that a patient’s values might differ from their own. Also, the student showed the capability to reflect on possible reasons behind the patient’s choices, characteristic of the cognitive aspect of empathy, and described the change in their thinking.

*With mental health patients I realized that I was pretty****helpless****in that situation. I don’t have much experience with mental health patients or how to deal with a psychotic patient, for example.*

The student described feelings of uncertainty and thoughts aroused after meeting a patient suffering from a disease they were not familiar with. Moreover, they showed an ability of self-reflection when describing their own lack of experience with psychiatric disorders.

Physician attitudes toward patients also gave rise to thoughts among the students.

*When I was present at a couple of doctors’ appointments I was impressed by how well they took the patient into consideration at all times.*

The student described feelings and considered the physicians’ attitudes and activities as positive examples. The student had an experience they could identify with as a future physician.

*What struck me the most was when a patient was explaining what the matter was, and the doctor would just turn from the computer screen and interrupt the patient to say something quite unrelated to the matter at hand. It was quite instructive to see how****patients can experience that they are not being listened to.****”*

*The doctor I shadowed in the emergency department had an extremely efficient approach to work.… Even though situations evolve very quickly in that setting and everything must be handled efficiently, I would still try to make sure that the time pressure would not be as evident to patients as it was in many of the cases I witnessed.*

The students described discomfort because of the physician’s behaviour. The haste bothered the student in both examples. In the first example, the student anticipated how the patient may have experienced the physician’s behaviour. The last example indicates that students considered alternative approaches are being more suitable in future events.

In these examples, students have to face their helplessness and uncertainty; additionally, their trust and belief in the future are being tested.

Learning diaries that processed students’ own inner experiences revealed uncertainty, previous experiences in personal life, and growing competence. While the majority of the students did not produce content about their own inner experiences, some were able to provide reflective or advanced reflective writing about their own inner experience during preclinical studies.

*Particularly at this stage, when the knowledge base is still so shaky, difficult patient cases mainly gave rise to****a sense of helplessness***.

*If I’m stressed about a three-day training period in the health centre where I have no responsibility for the patients and I’m really not yet expected to have much clinical competence, how will I manage in my first summer job and other stressful situations?*

*I noticed that I often felt a****sense of uncertainty****when the doctor asked for my opinion or my findings concerning the patient.*

*Watching the events in the emergency department this afternoon forced me to consider how I would manage in this kind of job under constant pressure and rush.*

*Clearly the greatest worry was when I realized the amount of medical knowledge that needs to be learned, that a person can really be ill in so many different ways, and I should be able to thrash out the correct alternative.*

In these examples, students have to face their helplessness and uncertainty, additionally their trust and believe in the future is being tested. Recognized feelings of uncertainty and described concerns regarding the demands of their future profession are questioned.

*My own attitude towards elderly and seriously ill persons has certainly been affected by experiences from my own circle of family and friends and witnessing palliative care. It was easy to remain calm and compassionate towards patients as well as their loved ones.*

The student reflected on their experiences during the training period and showed the capability to understand that one’s previous experiences in life have an effect in a professional context.

Moreover, students considered physician well-being to the psychological burden of work. Physician well-being mentioned in the learning diaries included reflection on the requirements of the profession and the physician’s ability to cope with psychically demanding situations. Additionally, the students discussed the coping and resilience of physicians. The following quotes are examples of the students’ reflections related to the well-being of physicians:

*Perhaps you put yourself too much in the patient’s shoes. Are you supposed to mull over difficult cases? Would that mean that being a doctor means that you are constantly worried? Having other things in life besides work helps separate patients’ worries from your own worries.*

The student described the thoughts a psychically demanding situation aroused in them. The student recognized that they easily share patients’ feelings and reflected on the difficulty of separating one’s own experience from the patient’s experience, which led to concerns regarding their future as a physician.

*I noticed that the most important thing that took place in me when meeting patients was a sense of empathy and compassion. When the patient felt bad, I sort of caught it, too. You don’t really know what to do in a situation like that.*

Here, the student described the feeling of *empathy*. They recognized how they tend to transfer patients’ feelings to themself. Also, they described feelings of uncertainty in these situations.

## Discussion

In this large qualitative study based on 142 s-year medical students’ reflections related to the general practice course the following themes arose: feelings towards end of life, demanding situations in practice, physician’s attitude to patient, student’s inner experiences, and physician’s well-being. Our novel finding was that students reflected on themes of patients’ alcohol/drug overuse, lifestyle diseases and psychosomatic symptoms. Additionally, psychically demanding situations and physician as a role model were recognized as themes. Three types of reflection capacities were detected: simple reporting, reflective writing and advanced reflective writing. Nearly all the learning diaries were reflective. Of note, some students demonstrated advanced reflective writing at this point in their studies. Advanced reflectivity was related to a deeper understanding of the reasons behind recognized feelings, thoughts, and attitudes.

This course is related to the primary care context in which patients are treated in various phases of their life spans and diseases. The medical curriculum is mainly focused on special care; therefore, understanding the holistic view of general practice is crucial. In Finland, all physicians work at least 9 months in primary care regardless of their future speciality. Therefore, medical students have an opportunity to become familiar with the practical side of the work at an early stage in their studies and have a versatile education regarding various diseases and conditions.

Reflective writing has been described as an effective tool from the very beginning of medical studies [16–18], and it seems to help students deal with different experiences and emotions during their first patient contacts [17]. Reflection has been investigated and utilized with respect to specific contexts, especially in terms of end-of-life matters and breaking bad news [16, 19, 20]. However, few studies have investigated reflective writing from a perspective similar to ours. We found one study that investigated situations related to feelings and thoughts that arise during first patient contacts [21]. According to prior studies, reflective writing seems related to similar themes, though other themes were identified as well. In the end, our interest was targeted at supporting the development of critical reflection, as it is a requirement for transformative learning and professional growth [9]. If reflective writing is used to maintain and enhance medical students’ reflective capacity as a part of medical education, it might be useful to include reflective practice, especially when corresponding themes (end of life, demanding situations in practice, physician’s attitude to patient, student’s inner experiences, physician’s well-being, patients’ alcohol/drug overuse, lifestyle diseases and psychosomatic symptoms) are present in the curriculum. Furthermore, numerous studies have highlighted the importance of mentoring [1, 2, 20, 22, 23]. Student willingness to learn from feedback is dependent on how it is given [20, 23]. Therefore, teachers also need education on reflecting as part of their teaching practice to be able to share the emotional content of student reflections [22].

Guided questions were formulated to be broad, so that the students were able to describe their thoughts in the learning diaries without any strict guidance. Themes, such as end-of-life and demanding situations in practice, arising from the learning diaries are applicable to all fields of the medical curriculum, and it is important to examine these themes in relation to professional growth.

Uncertainty was one of the themes regularly mentioned in the learning diaries. Uncertainty has been studied from several perspectives and with diverse approaches in medical literature, but less often specifically in terms of reflective writing [17]. Nevalainen et al. noticed that for students, reflective writing seemed an effective tool for both expressing and dealing with uncertainty [17]. According to our findings, uncertainty was not a separate theme but rather intertwined with other themes. In particular, uncertainty appeared in considerations regarding the requirements of their future profession and a physician’s ability to cope with psychically demanding situations. Even though a handful of students indicated high reflective capacity, keeping oneself separate from the patient’s experiences and emotions appeared to be quite difficult. Reflection supports this metacognitive skill, as metacognition defined as “Thinking about one’s own or another’s thinking or feeling” [24]. As second-year students, they did not have the responsibilities of a training physician, and the role of physician remained rather unfamiliar. Therefore, they seemed to share the experiences and emotions of the patients more easily (compared to the role of the physician). The capability to see things from the patient’s point of view is characteristic of the cognitive aspect of empathy [25]. Also, it is an essential skill in patient care to improve patient satisfaction and self-efficacy leading to better clinical outcomes [26–28]. Moreover, empathy comes with self-awareness [19] and the ability to keep oneself separate from another person’s emotions, which is the main difference between empathy and sympathy [25]. The future may seem frightening and demanding at this point of their studies, and it might remain so later if the ability to keep a distinct distance is lacking. Therefore, the development of empathic skills is necessary not only for the patient’s [26–28] but also for the physician’s well-being. Coping skills are essential in the profession.

Another regularly discussed theme was demanding situations in practice, including lifestyle diseases and alcohol/drug overuse. These themes challenged students’ thinking, and the majority considered lifestyle diseases and alcohol/drug abuse as consequences of patients’ own choices. However, some exhibited the capability to see possible reasons behind a patient’s undesirable behaviour. In some cases, they were even capable of understanding that the behaviour might be a self-protective response to an intolerable situation [29]. One student wrote: “[…] it’s not likely that anyone had wanted to choose a path that leads to alcohol or drug abuse. [… ] A lot of things have an effect on how people evolve and what happens to them.” This part of the vignette showed the student’s zone of proximal development within which their thinking can change [31]. Zone of proximal development is defined as the space where the student is able to perform, but only with support from a teacher or a peer with more knowledge or expertise [8]. The described change in one’s thinking is critical in preventing the physician’s attitudes and preconceptions from disturbing the physician-patient relationship and avoiding overdiagnosis. When willing to understand the complex and sometimes incomprehensible behaviour of the patient and to settle down as a physician in demanding situations, DSA offers a theoretical framework [15] DSA is a microanalytic method based on Mikahil Bakhtin’s theory of utterance and Vygotskyan theory of sign-mediated activity and is used to analyse utterances as described in Leiman [6]. As a research method, it comes close to Wortham’s tradition [15], which seeks signs of linguistic expressions; by analysing these expressions, a person’s attitudes towards the topic can be captured. We used DSA as a framework for our thinking, and it significantly affected how we approached and interpreted the students’ learning diaries and expressions.

Reflection has been defined in numerous ways [3, 4, 30, 31]. We chose to introduce specific theories, as our personal interpretation of reflection is close to those. Mainly, our interpretation relies on Mesirow’s notion of critical reflection, which highlights critical thinking about preconceptions and attitudes that can affect our actions and beliefs [9]. In this study, the three groups or levels of reflective capacity were formed based on the content of the learning diaries. Therefore, they do not meet the requirements of any introduced definition of reflection directly but rather describe the collected data, as the research was data-driven. However, the groups can be contrasted to known definitions. In addition to previously introduced theories, Wald et al. developed a theory-based tool in order to support medical students’ professional growth [13]. They achieved consensus on five levels of reflective capacity based on the theories of Schon [32], Boud and colleagues [33] Moon [34] and Mezirow [9] including two levels of nonreflective action (habitual and thoughtful), reflective level, critically reflective level and transformative learning. Critical reflection is a vital part of medical education, as it can be seen as a prerequisite for transformative learning [35]. The theory of transformative learning challenges previously established perspectives and produces new ways of thinking [36].

### Limitations and strengths

In qualitative research, the credibility of the study is dependent on the research process, which is described in the Methods section, including the research approach, the context and organization of the study, as well as the analysis of the material. The study has strengths and limitations. The high degree of participation by our selected population can be considered one of the strengths of this study. Keeping a learning diary was compulsory, which was, of course, an important reason for the high response rate. However, participation in the study was not mandatory. The diary was not a disconnected part of studies but played an important role in the course and supported learning. Therefore, the majority of the learning diaries were carefully written and provided high-quality material for qualitative analyses. Depending on the point of view, the guided questions may be considered a limitation or a strength. However, it is important to note that, without a doubt, facilitating affected the process. It is also noteworthy that one’s expressions are always addressed to an addressee [6]. In this case, students directed their words to the clinical lecturer, and this affected their writing, as well. Additionally, there might be factors which influence an individual’s style of written expression i.e. prior life experiences, vocabulary, first or second language usage, comfort and trust issues related to sharing inner thoughts with others especially teacher, preferred patterns of communications and verbal expression (the terse to effusive continuum), situational factors (such as time, other commitments and motivation), and learning preferences (i.e. concrete vs. abstract orientation). Furthermore, our population was representative of second-year students in the Medical Faculty of the University of xx. However, the study was carried out in a single university and describes how students reflect in a single Finnish medical faculty, which can be considered a limitation. However, the results are not only context-specific but instead deepen understanding of reflection as a phenomenon. Finally, analyses were conducted from the researchers’ viewpoint, and their experience and mindset inevitably had an impact on the analyses, which is characteristic of qualitative research, even though the study was data-driven.

## Conclusions

Professional growth requires the development of reflective capacity, which is essential for successful patient care and better clinical outcomes. Nearly all the learning diaries relating to the second-year general practice course of were reflective; especially demanding clinical situations and inner factors such as the professional’s point of view resulted in reflectivity. We believe that these themes must be discussed during the medical curriculum in general, and that they may also improve professional growth in relation to other specialities. Supporting students towards advanced reflectivity may lead to authenticity and meaningful patient–doctor relationships. Of note, some students demonstrated that they were already capable of advanced critical reflective writing. Advanced reflectivity seemed to relate with deeper understanding of the reasons behind feelings, thoughts and attitudes. If reflective writing is used to maintain and enhance the reflective capacity of medical students as part of medical education, it might be useful to include reflective practice when corresponding themes are presented in the curriculum.

## Data Availability

The data that support the findings of this study are available in Finnish from University of Oulu, but restrictions apply to the availability of these data, which were used under license for the current study, and so are not publicly available. Data are however available from the corresponding author, Ilona Mikkola upon reasonable request and with permission of University of Oulu.
